# The Epstein-Barr Virus Encoded BART miRNAs Potentiate Tumor Growth *In Vivo*

**DOI:** 10.1371/journal.ppat.1004561

**Published:** 2015-01-15

**Authors:** Jin Qiu, Pamela Smith, Leah Leahy, David A. Thorley-Lawson

**Affiliations:** 1 Department of Pathology, Tufts University School of Medicine, Boston, Massachusetts, United States of America; 2 Department of Hematology/Oncology, Tufts Medical Center, Boston, Massachusetts, United States of America; Tulane Health Sciences Center, UNITED STATES

## Abstract

The human herpes virus Epstein-Barr virus (EBV) latently infects and drives the proliferation of B lymphocytes in vitro and is associated with several forms of lymphoma and carcinoma *in vivo*. The virus encodes ~30 miRNAs in the BART region, the function of most of which remains elusive. Here we have used a new mouse xenograft model of EBV driven carcinomagenesis to demonstrate that the BART miRNAs potentiate tumor growth and development *in vivo*. No effect was seen on invasion or metastasis, and the growth promoting activity was not seen *in vitro. In vivo* tumor growth was not associated with the expression of specific BART miRNAs but with up regulation of all the BART miRNAs, consistent with previous observations that all the BART miRNAs are highly expressed in all of the EBV associated cancers. Based on these observations, we suggest that deregulated expression of the BART miRNAs potentiates tumor growth and represents a general mechanism behind EBV associated oncogenesis.

## Author Summary

Epstein-Barr virus is a herpes virus that persistently infects essentially every human being for life. It also has the ability to latently infect B lymphocytes and cause them to proliferate indefinitely in culture, and is associated with several forms of carcinoma and lymphoma. The virus contains genes for ~30 miRNAs in its BART region. The functions of these miRNAs are mostly unknown, but it is clear that they are not required to drive the growth of infected cells *in vitro*. We have shown previously, however, that these miRNAs are all highly expressed in the EBV associated cancers and that their expression is deregulated suggesting they may play a role *in vivo*. Until now, the significance of BART miRNAs to tumor development *in vivo* was unknown. Here we have used a mouse xenograft model to show that these miRNAs, while having little or no discernible effect on the growth of infected cells *in vitro*, potentiate the seeding and growth of EBV associated tumors *in vivo*.

## Introduction

Epstein-Barr virus (EBV) is a ubiquitous human herpes virus. It infects virtually every human being for life and in the overwhelming number of people this persistent infection is benign [1]. The virus achieves this by entering into a quiescent latent state within circulating, resting, memory B lymphocytes where no viral proteins are expressed [2, 3]. However, in order to achieve this state the virus needs to latently infect naïve B lymphocytes and drive them to become activated proliferating lymphoblasts (growth transcription program\Latency III) so that they can then differentiate through the germinal center to become latently infected, resting memory B cells. This ability to drive B cell proliferation has long been believed to explain why EBV is associated with several forms of cancer, including lymphomas (Burkitt’s (BL) and Hodgkin’s (HD)) and carcinomas (nasopharyngeal (NPC) and gastric (GaCa)) [1, 4]. However, a major inconsistency with this idea was uncovered when detailed analysis of viral latent protein expression was performed on infected cells *in vivo* and in tumor cells. These studies revealed that the tumors do not express the full panoply of potentially oncogenic, growth promoting, EBV latent proteins found in the growth program. Rather, the EBV gene expression profiles seen in the tumors reflects those found at specific stages of B cell infection *in vivo* [4]. This finding has led to the proposal that the tumors arise directly from these infected B cell types. In the most extreme case (BL), only one viral protein is expressed, EBNA1 [5, 6] which is the gene expression pattern characteristic of latently infected memory B cells [7] when they divide. These counterintuitive observations strongly imply that the virus has evolved to minimize the oncogenic risk posed by the growth program/Latency III latent proteins, which otherwise would threaten the host within which the virus persists.

On the other hand, it is well known that the EBV episome, or plasmids derived from it, will be rapidly lost if there is no selective advantage to their retention [8–11]. The persistence of the viral episome within the tumors therefore implies that the virus must be contributing to the growth and/or survival of the tumors [8, 12–17]. Since most of the growth promoting EBV latent proteins are absent, the question arises as to what the virus is contributing to the tumors that causes the viral episome to be retained. Recently, attention has become focused on abundant non-coding EBV RNAs [18–23]. These include ~40 miRNAs, four of which are encoded from the BHRF region and the remainder from the BART region. We have shown that all of the BART miRNAs are highly expressed in all of the tumor types, especially the carcinomas [22, 24]. This includes a subset of the BART miRNAs whose expression *in vivo* is specifically associated only with the Latency III growth program (Latency III associated BART miRNAs) and thus, would not be expected to be associated with the viral latency programs found in the tumors (Latency I or II). To date, this represents the only example of inappropriate expression of Latency III growth program genes in the tumors and suggests that these miRNAs may play a role in tumor development.

Although the functions of most of the BART miRNAs remains unresolved several *in vitro* studies have suggested that they may contribute to oncogenesis. BL-derived cell lines with constitutive expression of BART miRNAs are able to inhibit apoptosis induced by the loss of EBV *in vitro*, implicating a survival role for BART miRNAs in BLs [25]. BART 5 represses the p53 up-regulated modulator of apoptosis (PUMA), thereby protecting EBV-infected cells from virally-induced apoptosis and in addition high expression of BART 5 correlates with the low abundance of PUMA in NPC tissues [26]. BART 13* likely plays an anti-apoptotic role by targeting the Wnt-signalling enhancer CAPRIN2 [27], Bart 3* suppresses the DICE1 tumor suppressor to promote cellular growth [28] and BART Cluster 1 miRNAs are able to suppress the Bcl-2 interacting mediator of cell death (Bim), thus protecting cells from apoptosis [29]. However, until now no data in support of a role for the EBV BART miRNAs in tumorigenesis *in vivo* has been presented. Since it is not possible to study the tumors in situ in humans, we developed a mouse model of one (NPC) [30]. In this paper we use this *in vivo* model to demonstrate that the miRNAs confer a significant growth advantage to EBV associated tumors *in vivo*.

## Results

### Up regulation of EBV BART miRNAs in nasopharyngeal carcinoma *in vivo*

We have previously reported an orthotopic mouse model which faithfully recapitulates locally invasive and metastatic EBV-positive nasopharyngeal carcinoma (NPC) [30]. In this model, luciferase-tagged C666–1 cells were injected into the nasopharyngeal epithelium of the highly immune deficient mouse strain NOD.Cg-PrkdcscidIl2rgtm1Wjl/SzJ (NSG). To gain insight into the possible role of EBV BART miRNAs in tumorigenesis we wished to ascertain if tumor growth and/or metastasis in this model was associated with preferential expression of specific BART miRNAs. Therefore, we examined the BART miRNA expression profiles of C666–1 cells before and after inoculation and growth as tumors in the NSG mouse model and then again after the tumors had been explanted and grown *in vitro*. A complete description of the parental lines, tumors, and tumor explant cultures developed and used in this study is given in S1 and S2 Tables. To profile the BART miRNAs we employed a PCR based assay that we have characterized in detail previously [24]. This assay will detect ≤10 copies of each mRNA with a linear response up to ≥10^8^ copies. It has the advantage that it allows the quantitative profiling of the expression of a large number of miRNAs from small tissue samples that otherwise would be impossible to study, such as human tissue biopsies and the moue tumors studied here.

**Figure 1:**
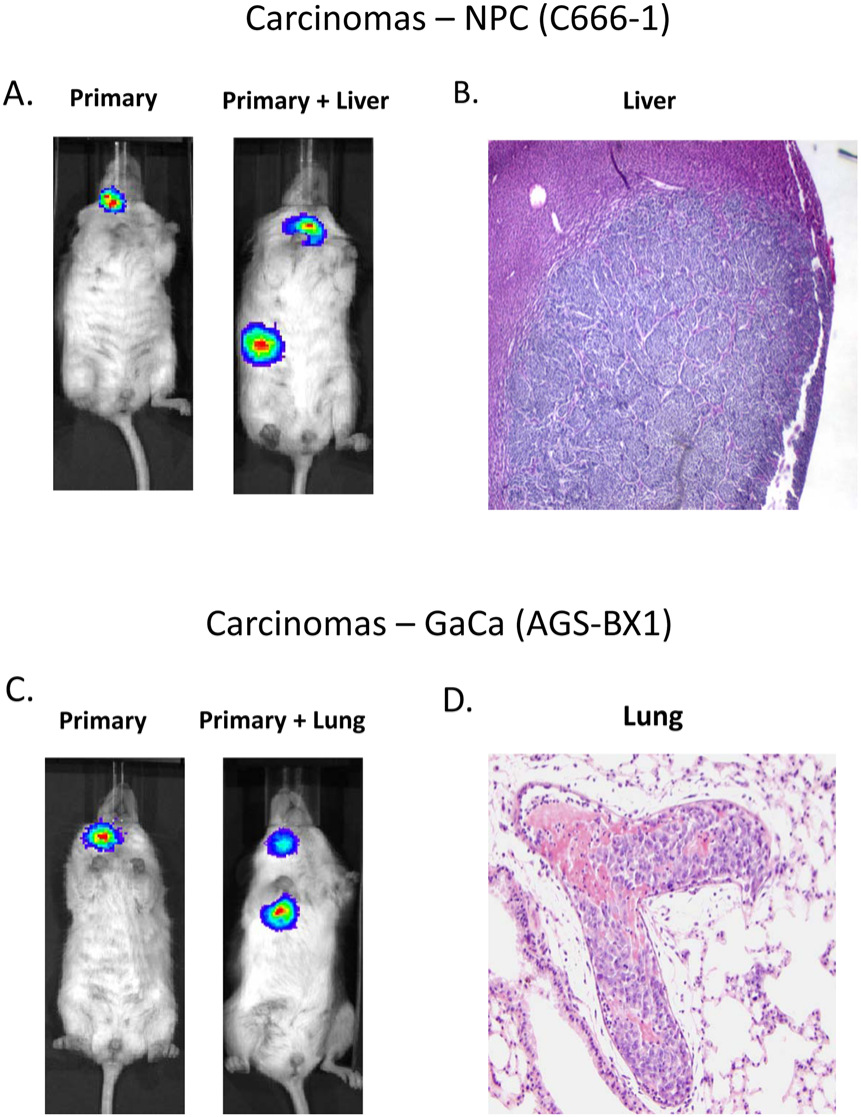
Mouse models of EBV positive carcinoma. Cells from the luciferase tagged NPC line C666–1 and GaCa line AGS-BX1 were injected into the nasopharyngeal epithelium of NOD.Cg-PrkdcscidIl2rgtm1Wjl/SzJ mice and tumor growth regularly monitored. A. Vital scans demonstrating the growth of primary tumors and metastases for C666–1 NPC cells. B. Typical histology of metastases from C666–1 NPC cells. C. Vital scans demonstrating the growth of primary tumors and metastases for AGS-BX1 GaCa cells. D. Typical histology of metastases from AGS-BX1 GaCa cells.

**Figure 2:**
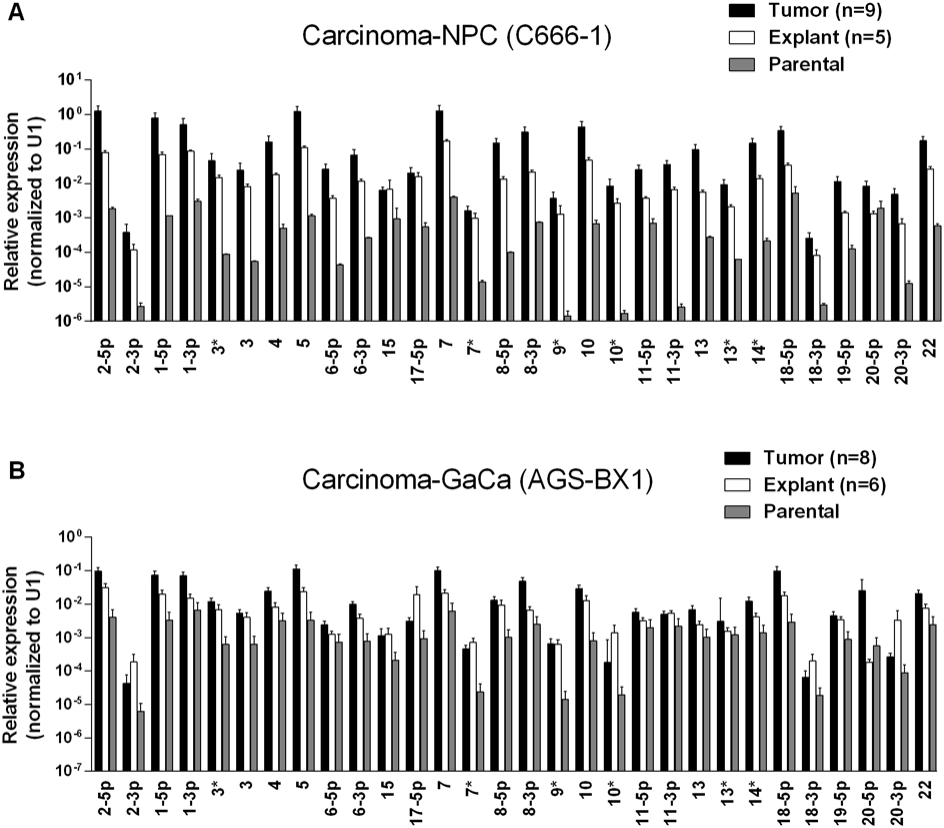
BART miRNA expression is up regulated when carcinoma cells are grown *in vivo*. A. The expression profile of the BART miRNAs in the C666–1 parental cell line (grey), *in vivo* tumors (black) and *in vitro* explants derived from the tumors (white). B. The expression profile of the BART miRNAs in the AGS-BX1 GaCa parental cell line (grey), *in vivo* tumors (black) and *in vitro* explants derived from the tumors (white). N.B. All BART miRNA levels are expressed relative to the ubiquitous small cellular RNA U1.

Fig. 1A and B show examples of C666–1 derived tumors grown in the NSG mouse model. The miRNA profiles of the cell lines before, during and after growth as tumors are shown in Fig. 2A. We did not observe specific changes in individual miRNAs rather all of the BART miRNAs were significantly up regulated when the cells grew as tumors *in vivo* compared to the parental lines (p<0.001). When the tumors were explanted for culture *in vitro* to become cell lines, the miRNA expression declined again to that of the parental line. However, this decline was fully reversible since upon reinjection back into the mice, the miRNA level again increased. In all, this process was reiterated three times with the same result, up regulation *in vivo* and down regulation *in vitro* (not shown). This suggests that increased expression of the BART miRNAs may confer a growth advantage to the tumor cells *in vivo*. Two of the three BHRF1 miRNAs also showed elevated copy numbers in the tumors although their expression levels were modest when compared to the BART miRNAs (not shown).

**Figure 3:**
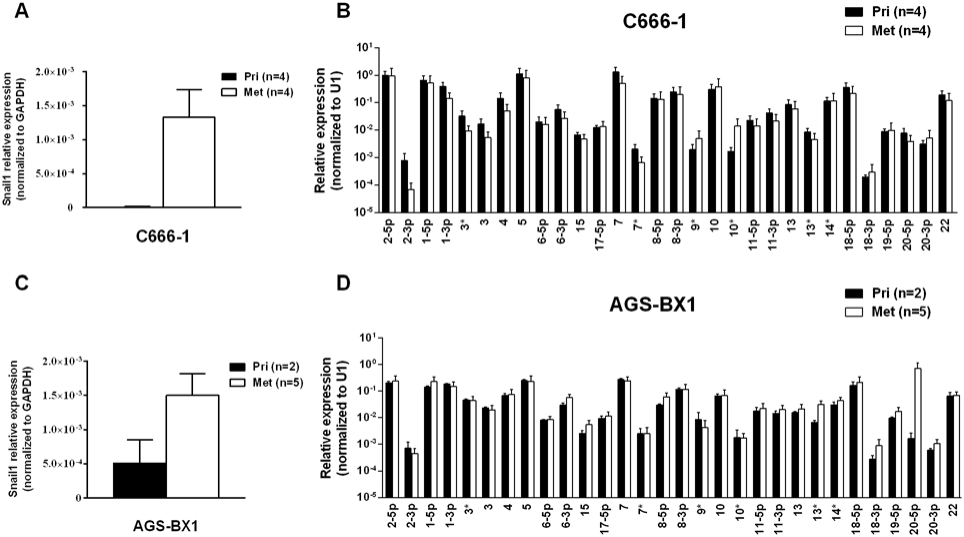
BART miRNA expression does not change in metastasis. A. and C. mRNA expression level of the metastasis associated transcription factor Snail in primary tumors and metastases from C666–1 (A) and ABS-BX1 (C) cells. B. and D. Expression profile of the BART miRNAs in C666–1 (B) and AGS-BX1 (D) primary tumors (black) and metastases (white). N.B. All BART miRNA levels are expressed relative to the ubiquitous small cellular RNA U1.

Metastasis is generally characterized by expression of the signature protein Snail a key transcriptional factor in epithelial mesenchymal transition that is known to promote metastasis [31]. As expected, Snail expression was greatly increased in the C666–1 metastases compared to the primary tumors (Fig. 3A). However, when we compared the BART miRNA expression profiles of primary tumors with metastasis they were indistinguishable (Fig. 3B). Indeed we have developed a highly metastatic derivative of the C666–1 tumor with a 100% metastasis rate in mice and even these cells showed no variation in BART miRNA expression (not shown). Thus, this analysis did not detect a correlation between EBV BART miRNA expression and metastasis.

### Up regulation of EBV BART miRNAs in gastric carcinoma *in vivo*

We wished to confirm these findings in a second carcinoma model therefore we chose to study the gastric carcinoma (GaCa) cell line AGS-BX1. Successful engraftment of immunosuppressed mice with this cell line has not been reported previously. Consistent with this, mice injected subcutaneously with 10^7^ AGS-BX1 cells failed to develop tumors (not shown). Interestingly though, 4 out of 10 mice injected in the nasopharyngeal epithelium (I.N.) grew tumors, with metastatic dissemination being observed in 3 of the mice (Fig. 1 C and D) suggesting that this is also a good model to study GaCa *in vivo*. We profiled the BART miRNA expression levels in the parental line, tumors and explants and observed similar results to those seen with the NPC model (Fig. 2B). Although not as large as the NPC line, the increase in BART miRNA expression in the tumors was nevertheless substantial when compared to the parental line (p<0.001). However, again no significant difference was seen between the primaries and metastases (Fig. 3 C and D) and the expression level decreased when the tumors were grown *in vitro*. The extent of reduction was not as dramatic as that seen with NPC and had not returned to the levels seen in the parental line at the time the cells were tested. This likely reflects the fact that the AGS-BX1 explant lines were grown for a much shorter time *in vitro* then the NPC lines and may not have had sufficient time to fully reduce their expression level.

### Specificity of the BART miRNA up regulation in tumors

**Figure 4:**
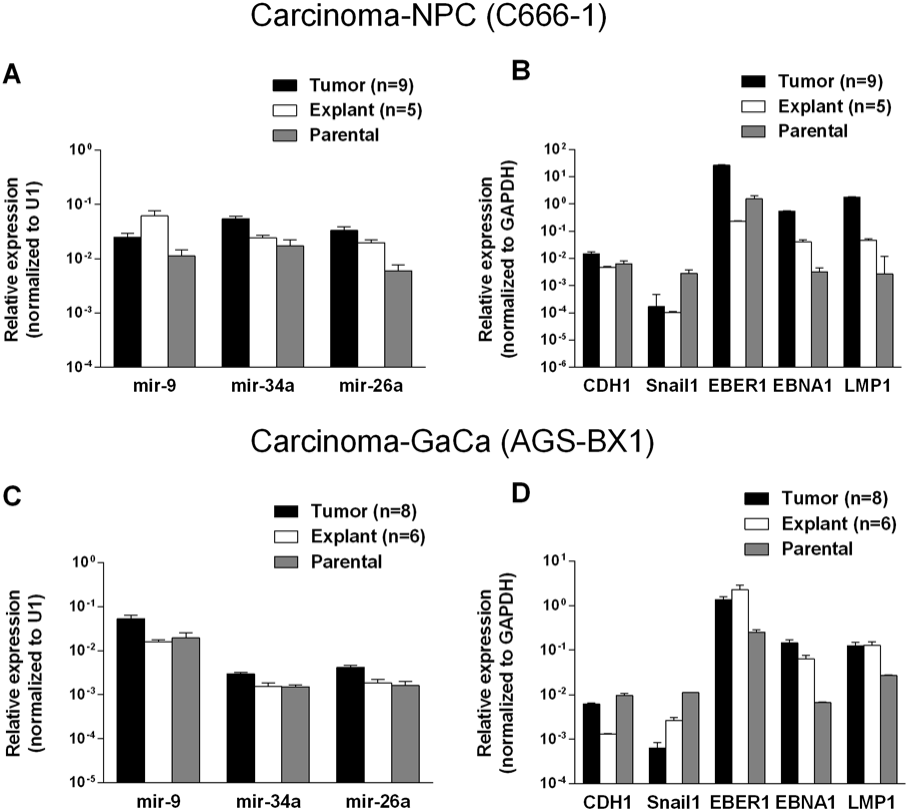
Changes in cellular and viral gene expression in the tumors. A. and C. Expression of three cell miRNAs in the parental line (grey) tumors (black) and tumor derived *in vitro* lines (white) for the C666–1 NPC cells (A) and for the AGS-BX1 GaCa cells (C). B. and D. Expression of cellular and EBV encoded mRNAs in the parental line (grey) tumors (black) and tumor derived *in vitro* lines (grey) for the C666–1 NPC cells (B) and for the AGS-BX1 GaCa cells (C).

To discover if the up regulation of the BART miRNAs was specific to the viral miRNAs or represented a more general phenomenon we first examined the expression of three cellular miRNAs mir-9, mir-34a and mir-26a. As seen in Figs. 4 A and C all three cellular miRNAs tested were expressed at similar levels in the parental lines, tumors and in the explanted tumor cells grown *in vitro*. A trend was observed as with the BART miRNAs in that levels were slightly higher in the tumors and tended to decrease again in the explants. However, these changes were not consistent (mir-9 was mostly highly expressed in the NPC explants) and did not always achieve statistical significance. In particular changes in the GaCa cells never achieved significance. Thus, unlike the BART miRNAs, the cellular miRNA increases were small and not reproducible between different cell types. This study confirms that the up regulation we have observed in BART miRNA expression in vivo was not a consequence of a global increase in the production of all miRNAs.

We also tested mRNA levels for two cellular genes that are associated with epithelial mesenchymal transition, CDH1 and Snail [32, 33] (Fig. 4 B and D). While the levels of CDH1 remained unchanged, Snail levels actually decreased in the tumors compared to the parental or explanted tumor cells grown *in vitro* for both NPC and GaCa. These results demonstrate that the increased expression of the BART miRNAs was not a consequence of a general up regulation in gene expression.

Lastly, we checked the mRNA levels of EB viral latent genes including the small viral RNA EBER1 (Fig. 4 B and D). The levels of all three were higher in the tumors for both the NPC and the GaCa cells when compared to the parental lines. However, the levels of the EBNA1 and LMP1 transcripts were strikingly up regulated for the NPC tumors suggesting that these latent proteins might also play a role in NPC tumor growth.

We conclude that there is an increase in the expression of the BART miRNAs when EBV positive carcinoma cells are grown as tumors *in vivo* and this is reversed upon re-culture *in vitro*. However, we saw no consistent changes in BART expression when tumors underwent metastasis. This suggests that the BART miRNAs may confer a significant growth advantage to EBV positive tumor cells *in vivo* that is not reiterated *in vitro*.

### The up regulation of EBV BART miRNAs *in vivo* is not carcinoma specific

**Figure 5:**
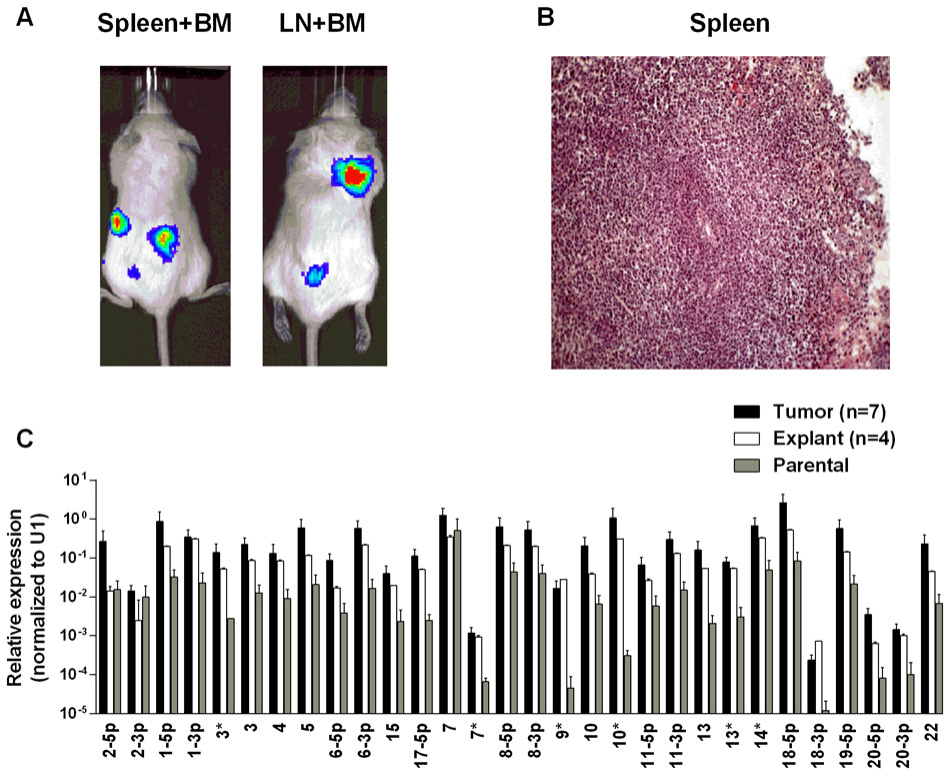
BART miRNA expression is up regulated when lymphoma cells grow *in vivo*. Cells from the luciferase tagged BL line BL36 were injected IV into the tail vein of NOD.Cg-PrkdcscidIl2rgtm1Wjl/SzJ mice and tumor growth regularly monitored. A. Vital scans demonstrating the growth of primary tumors and metastases for BL36 cells. B. Typical histology of metastases from BL36 tumors. C. The expression profile of the BART miRNAs in the BL36 parental cell line (grey), *in vivo* tumors (black) and *in vitro* lines derived from the tumors (white). N.B. All BART miRNA levels are expressed relative to the ubiquitous small cellular RNA U1.

To test whether the up regulation of BART miRNAs is tissue specific, i.e. restricted to the carcinomas, we evaluated their expression in a B cell lymphoma. Single cell suspensions of the luciferase tagged EBV-positive BL (BL36) cells could not be successfully injected into the nasopharyngeal epithelial tissue of NSG mice, therefore we injected them intravenously into the tail vein. Lymphoma developed in all injected animals (Fig. 5A and B). For a detailed description and account of the tumors used in this study see S1 and S2 Tables. Upon profiling the BART miRNA expression pattern we saw a similar result to that obtained with the carcinomas. The expression level of all the BART miRNAs was up regulated (p<0.001), when compared with the parental line (Fig. 5C). As with the carcinomas this effect was reversible when the tumors were re-cultured *in vitro*.

### EBV BART miRNAs potentiate tumor growth *in vivo*

**Figure 6:**
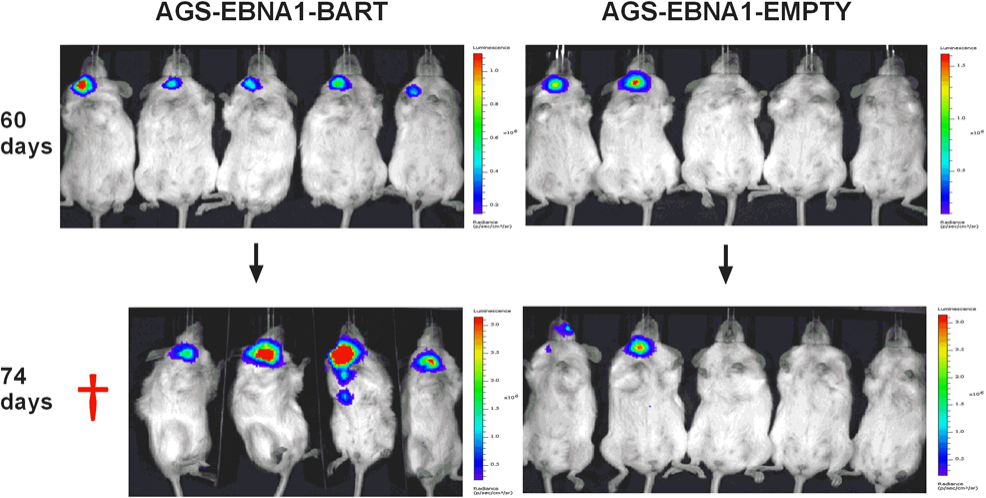
The BART miRNAs potentiate tumor growth *in vivo*. Cells from the luciferase tagged GaCa cell line AGS expressing either an empty vector (AGS-EBNA1-EMPTY) or a vector with all of the BART miRNAs (AGS-EBNA1-BART) were injected into the nasopharyngeal epithelium of NOD.Cg-PrkdcscidIl2rgtm1Wjl/SzJ mice and tumor growth regularly monitored. Scans of mice for luciferase expressing tumor cells at day 60 and 74 post-inoculation are shown. The red cross denotes a mouse that had already been sacrificed prior to imaging.

We have reported here an increased expression of the BART miNAs in EBV associated tumors *in vivo* that is not due to a general increase in cellular transcription but may be associated with increases in expression levels of other viral latent genes. To assess the contribution of the BART miRNAs alone to tumor development we took advantage of an EBV negative derivative (AGS) of the AGS-BX1 GaCa cell line. Luciferase tagged AGS cells were transfected with either an empty oriP/EBNA1 vector (AGS-EBNA1-EMPTY) or one that expresses all of the BART miRNAs (AGS-EBNA1-BART) [25]. These cells were then inoculated into the nasopharynx of NSG mice and tumor growth and metastasis *in vivo* were monitored (Fig. 6). After 60 days, 2 of 5 control mice had developed small but detectable tumors. Strikingly however, all 5 mice with AGS-EBNA1-BART (100% incidence) had malignancies, indicating that the BART miRNAs promote tumor formation. Furthermore, the BART-expressing tumors appeared to be more aggressive, as these mice deteriorated rapidly, developing large tumors and requiring sacrifice beginning at day 74. In contrast, by this time, still only two control mice showed detectable tumors that remained small and the mice appeared healthy. Thus, BART miRNAs enhanced both the rate of tumor formation (p = 0.03 by Fishers exact test at day 74) and tumor progression/fatality (p = 0.03 by Fishers exact test at day 88).

**Figure 7:**
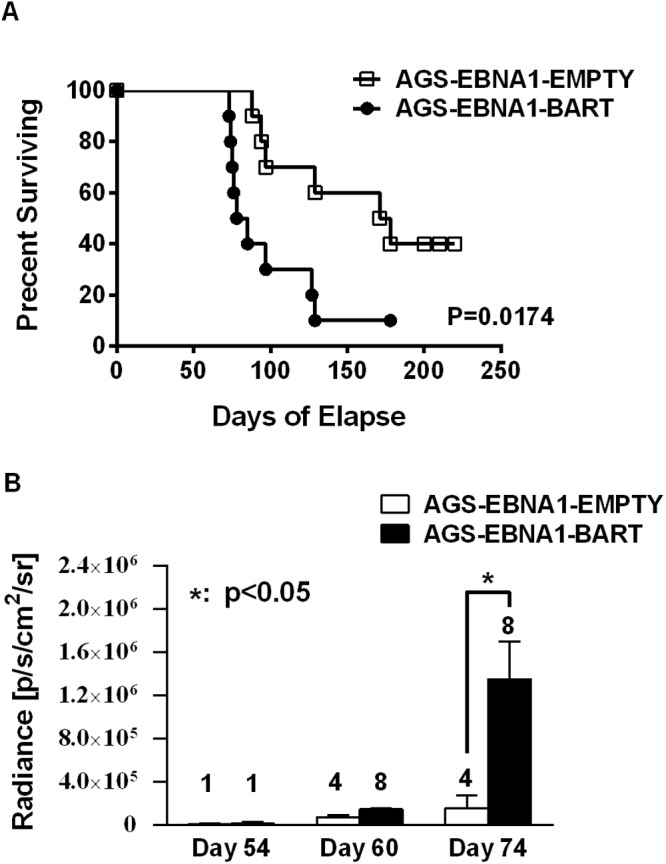
The BART miRNAs potentiate tumor growth *in vivo*. A. Kaplan-Meier survival analysis of the combined data from two experiments such as the one shown in Fig. 6. B. The tumor burden of mice assessed on the brightness of luciferase emission expressed as (p/s/cm2/sr). The number of surviving tumor bearing mice available for analysis at the given time point is shown above each bar. The data show the combined results from two experiments such as the one shown in Fig. 6 (p = 0.03 at day 74). N.B Since AGS-EBNA1-EMPTY and AGS-EBNA1-BART cells were derived from the same luciferase tagged AGS cell line their luciferase emission per cell was the same.

Kaplan Meier analysis of the complete data set combined from two such experiments confirmed that the AGS-EBNA1-BART mice exhibited a significantly higher overall level and rate of mortality relative to the AGS-EBNA1-EMPTY mice (Fig. 7A, p = 0.017). The more aggressive nature of the BART+ tumors was confirmed by measurement of the tumor burden in the two populations of mice. The combined data from two experiments is shown in Fig. 7B. No differences in tumor burden (measured as luciferase radiance) were evident between the two groups on day 60, but subsequently differences became apparent as the tumors progressed. By day 74, when most of the AGS-EBNA1-BART mice needed to be sacrificed, the tumor burden was approximately 10-fold higher than that of the AGS-EBNA1-EMPTY (~1.4×10^6^ p/s/cm2/sr in BART+ vs 1.5×10^5^ p/s/cm2/sr in EMPTY), suggesting that the BART miRNAs promote tumor growth *in vivo*. By taking measurements of luciferase emission from the same tumors over time it was possible to estimate their relative growth rates. This estimate suggested that the AGS-EBNA1-BART tumors were growing approximately twice as fast as the AGS-EBNA1-EMPTY tumors. We conclude that the BART miRNAs strongly promote tumor growth *in vivo*.

### The BART miRNAs promote tumor cell growth

**Figure 8:**
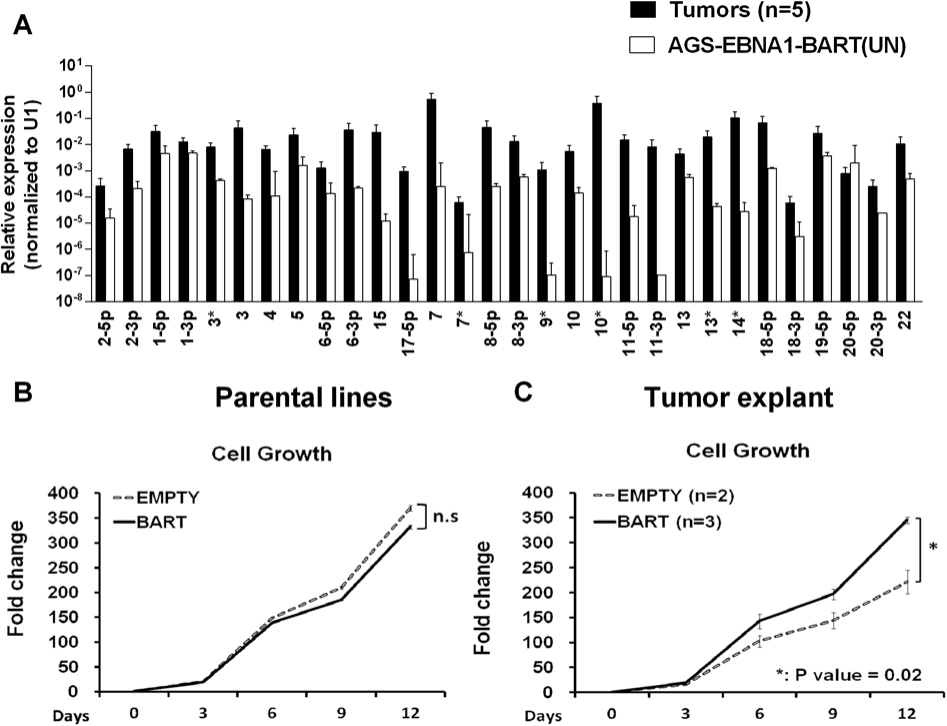
*In vitro* analysis of newly explanted tumor cells and their parental *in vitro* lines. A. Expression profile of the BART miRNAs in the parental AGS-EBNA1-BART cell line (white) and the *in vivo* grown tumors (black). B. and C. The proliferation in culture of the parental (B) and newly explanted tumor (C) cells. Proliferation was measured as described in Methods. The experiment was performed in triplicate on the number of independent tumors indicated. The value is the average ± standard deviation. n.s. represents a not statistically significant difference (p > 0.05). * represents a p < 0.05).

When we measured the BART miRNA expression profiles in the parental AGS-EBNA1-BART line and the tumors we observed the same effect as with the EBV positive tumor lines. We did not observe differential expression of the BART miRNAs rather all of the BART miRNAs were elevated in the *in vivo* tumors (Fig. 8A). For a list of all tumors and cells used for profiling see S1 and S2 Tables. This result confirms that BART miRNA up regulation *in vivo* is specific and not simply an indirect consequence of a general increase in viral latent gene expression.

Insight into the mechanism by which the BART miRNAs potentiated tumor growth was provided by *in vitro* analysis of AGS-EBNA1-BART and AGS-EBNA1-EMPTY tumors immediately after explant. Both AGS-EBNA1-EMPTY and AGS-EBNA1-BART parental lines grew at identical rates in culture prior to inoculation (Fig. 8B). However, after explant the AGS-EBNA1-BART tumor cells grew significantly faster (~60% faster) than the AGS-EBNA1-EMPTY tumor cells (Fig. 8C). AGS-EBNA1-BART tumors cells also showed a slightly higher rate of colony formation after explantation (S1A and B Fig.). Surprisingly, in light of recent studies on the effects of BART miRNAs on AGS cells *in vitro* [29] we observed no difference in the levels of apoptosis between the BART positive and BART negative parental lines when treated with the DNA damaging agent etoposide (S1C Fig.). We did observe a small decrease in sensitivity for the AGS-EBNA1-BART tumor explants when compared to AGS-EBNA1-EMPTY tumors. (8% specific reduction in apoptotic cells) (S1D Fig.) Although the differences in colony formation and apoptosis were statistically significant they were small suggesting that the main effect of the BART miRNAs is to provide a growth advantage to the tumors that is seen *in vivo* and detected upon immediate culture of the explants but lost after long term *in vitro* culture

These data suggests that the up regulated expression of the BART miRNAs confers a selective advantage to tumor growth *in vivo* that is not seen *in vitro*. Furthermore, they demonstrate a direct link between the up regulation of the BART miRNAs, that we have consistently observed with tumors *in vivo*, and enhanced tumor growth.

### BART expressing plasmids are preferentially retained in tumors

Plasmids constructed from an EBV oriP/EBNA1 vector do not integrate into the host genome when transfected into cells, but instead persist episomally [11]. However, these episomes are rapidly lost in the absence of selection pressure for their retention [9, 11, 34]. In the presence of drug selection the parental lines maintained ~450–500 copies of the EBNA1-BART and EBNA1-EMPTY plasmids per cell and as expected, both plasmids were lost when the lines were cultured in the absence of drug selection (not shown). This is consistent with our conclusion that the BART miRNAs do not convey a growth advantage to cells *in vitro*. Analysis of the plasmid copy numbers from a collection of the tumors (Table 1) revealed that the plasmid had been lost from the AGS EBNA1-EMPTY tumors (n = 4), which had an average copy number of less than 1 per cell (0.1±0.1 episomes per cell). However, the AGS EBNA1-BART tumors (n = 6), retained an average of 8.9±3.5 episome copies per cell (p = <0.001) which were only lost when the tumors were explanted and grown again in culture. Thus, the lack of drug selection *in vivo*, caused a precipitous drop in plasmid copy number for both cell types resulting in the loss of all the EBNA1-EMPTY plasmids whereas the EBNA1-BART plasmids were stably retained at around 5–10 copies per cell. In parallel, the level of BART miRNA expression increased. Therefore, amplification of the episome copy number cannot explain the increased expression of the miRNAs in the tumors. This result demonstrates that expression of the BART miRNAs confers a selective advantage to the tumor cells *in vivo* that alone is sufficient to ensure retention of the plasmid that expresses them and confirms that this advantage applies to *in vivo* but not *in vitro* growth.

**Table 1:** Plasmid copy number in tumors with or without BART miRNA expression.

	**AGS-EBNA1-BART**						**AGS-EBNA1-EMPTY**			
Plasmid Copy Number/Cell	7.2	8.9	10.7	2.4	10.5	13.9	0.02	0.02	0.1	0.3
Average	8.9±3.5*						0.1±0.1*			

* p value = <0.001

We conclude that the EBV BART miRNAs do not provide a detectable growth or survival advantage when the cells expressing them are grown *in vitro*. However, *in vivo* they provide a pronounced advantage to the tumors specifically causing them to be seeded more efficiently and grow faster and more aggressively.

### BART miRNA expression does not affect invasion/metastasis

**Figure 9:**
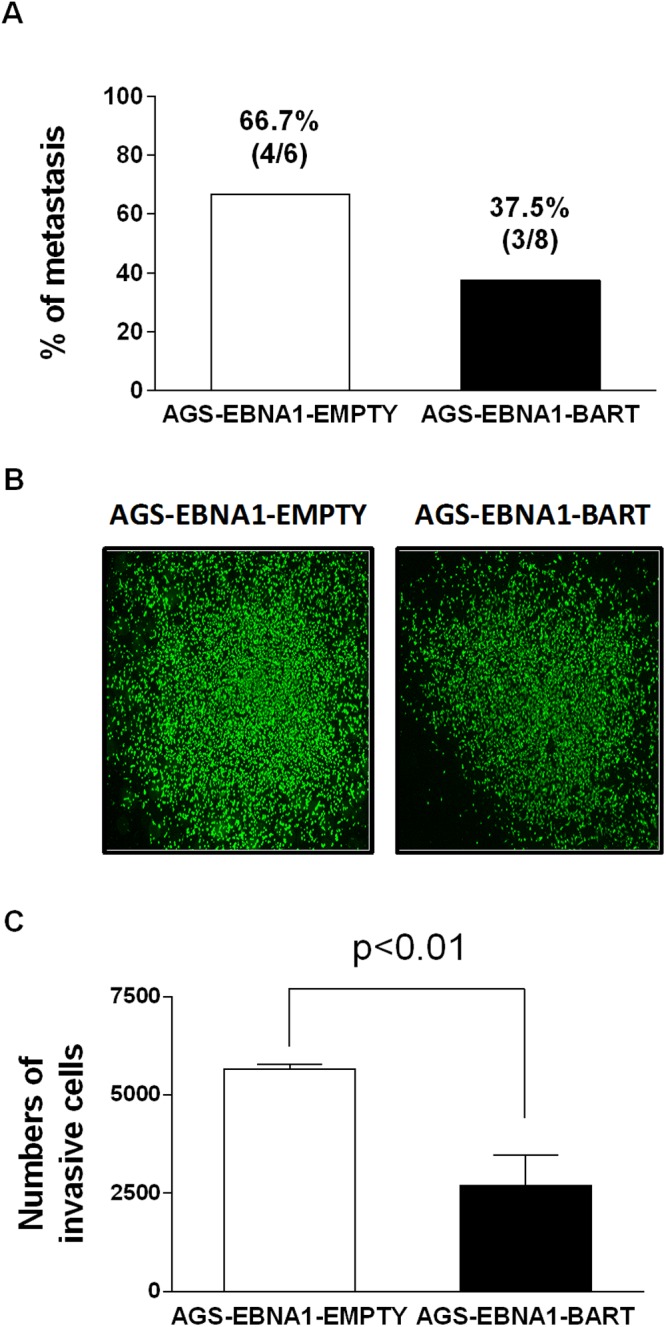
BART miRNA expression does not affect metastasis or invasion. A. Rate of metastases in mice injected with AGS-EBNA1-EMPTY (white) or AGS-EBNA1-BART (black) cells. The rate is expressed as total number of mice with metastases over the total with tumors. The percentage and actual numbers of mice are shown above each bar. B. and C. Matrigel invasion assay of AGS-EBNA1-EMPTY (white) or AGS-EBNA1-BART (black) cells. B. Images of cells that had invaded. C. quantitation of the number of migrating cells. For details of the assay see Methods.

Next we asked if expression of the EBV miRNAs in AGS cells would provide for a higher rate of metastasis. For mice receiving AGS-EBNA1-EMPTY cells, 4 out of 6 mice with tumors (66.7%) developed metastases (Fig. 9A), whereas only 3 out of 8 mice with AGS-EBNA1-BART tumors (37.5%) developed metastases. Although these numbers do not achieve statistical significance, they are consistent with a trend that the BART miRNAs do not exacerbate metastasis and might actually impede it.

Lastly, we asked if the BART miRNAs could confer a more invasive phenotype to the tumor cells, as cell invasion is a critical step in metastasis. To test this we performed trans-well Matrigel invasion assays comparing AGS-EBNA1-EMPTY cells with AGS-EBNA1-BART cells. In this assay, the ability of cells to invade through an extracellular matrix of a Matrigel-coated porous membrane in response to chemoattractants is assessed. After a 24 hour incubation, the AGS-EBNA1-BART cells demonstrated a slightly less invasive phenotype than did the AGS-EBNA1-EMPTY cells, as the absolute cell numbers of AGS-EBNA1-EMPTY on the trans-side (migrated cells) were around 2-fold higher than that of AGS-EBNA1-BART (Fig. 9B and C), suggesting that EBV BART miRNAs may also have a negative effect on invasion. We conclude that our experiments provide no evidence for BART miRNAs contributing positively to invasion and/or metastasis.

## Discussion

In this paper we have demonstrated that the EBV encoded BART miRNAs confer a selective growth advantage to EBV positive tumor cells *in vivo*. This growth promotion occurs in parallel with an up regulation in the expression of all the BARTs. Furthermore, elevated expression of the BART miRNAs *in vivo* was observed for every EBV positive tumor type we tested. This suggests that the BART miRNAs confer a growth advantage to all EBV positive tumors.

The lack of a readily accessible and manipulable animal model of EBV infection and tumorigenesis means that most studies on the virus’s biology must be conducted *in vitro*. Thus to date, all studies on BART miRNA functions have been performed *in vitro* and have yet to be verified or shown to be biologically meaningful in an *in vivo* setting. Lacking evidence from humans, mouse models can provide an alternate approach to investigate the role of EBV encoded genes *in vivo*. Inoculation of immunocompromised mice (e.g. severe combined immune deficiency or SCID mice) with EBV-positive tumor-derived cell lines has been extensively used to study the role of the virus in tumorigenesis [30, 35–41]. However, these models rarely, if ever, recapitulate key aspects of the tumors behavior, such as invasion and metastasis. We have previously described a mouse model that accurately reproduces locally invasive and metastatic EBV-positive carcinoma [30] and have applied this model here to study the role of the EBV BART miRNAs in tumor development. We have shown that expression of the BART miRNAs resulted in more efficient tumor seeding, larger tumors and higher and more rapid mortality. Indeed the tumor burden in the BART+ mouse group was ~10 times greater after 74 days of growth *in vivo* than with the BART- group. Furthermore, the newly explanted tumors grew 60% faster in culture if they expressed the BART miRNAs, an increase in growth rate sufficient to account for the differences seen *in vivo*. However, this effect was not sustained in long term cultures. Although we cannot definitely rule out other activities for the miRNAs in tumor development we saw only minimal changes in other functions we assayed including colony formation and resistance to apoptosis. For example, we confirmed the work of others that the BART miRNAs had an anti-apoptotic effect [25–29], However, this effect was very modest in our system and only detectable after *in vivo* growth. Contrary to previous findings [29] we did not observe any effect of the BART miRNAs on apoptosis when expressed in AGS carcinoma cells *in vitro*.

Independent proof that expression of the BART miRNAs conferred a selective growth advantage *in vivo* was provided by the observations on plasmid retention. OriP based plasmids do not integrate, but persist as episomes that are lost unless selective pressure is applied for their retention [11]. The retention of the EBV episome has been interpreted as compelling evidence that the virus is involved in the development in EBV positive tumors [12–15, 17]. Thus our observation that the BART expressing oriP plasmids (EBNA1-BART) were similarly retained in the tumor cells *in vivo*, whereas the empty vector (EBNA1-EMPTY) was lost, supports our conclusion that the BART miRNAs contribute to tumor growth. It follows that expression of the BART miRNAs is responsible in part for the retention of EBV in human tumors. Confirmation that our observation was an *in vivo* phenomenon came from the finding that both plasmids were lost from the cells in culture. These plasmids were not only selectively retained *in vivo*, but the BART miRNAs were expressed in the tumors at a much higher level than in the parental line grown *in vitro*, demonstrating a direct link between high BART copy number and rapid tumor growth. It is interesting to note that the EBNA1-EMPTY plasmid was lost *in vivo*, even though it expresses the viral tethering protein EBNA1 and we did not see an increase in EBNA1 expression when the AGS-EBNA1-BART in vitro lines grew as tumors in the mice (not shown) Together these observations suggest that EBNA1 does not provide a detectable advantage to tumor growth in our system. This raises the provocative question of whether it will be possible to develop a therapy that can be applied to all EBV tumors, based on ridding the tumors of the viral episome and therefore the virus, by silencing the BART miRNAs.

It is known that the BART miRNAs are not essential for the *in vitro* transformation and growth of B cells [42, 43]. This is consistent with our conclusion that the growth enhancement function of the BARTs only applies *in vivo*. However, deciphering the BART miRNAs responsible for the effect we have observed and the target genes of those miRNAs will be extremely challenging. Our observation that the copy number of all the BART miRNAs increases *in vivo* for all the tumor types we have studied here and our previous finding that all of the BART miRNAs are highly expressed in biopsies from all types of EBV associated tumors raises the possibility that all of the BART miRNAs may be contributing to tumor growth.

Despite the extensive effort of many laboratories there remain only a few well characterized targets of the BART miRNAs [44]. Of these anti-apoptotic targets predominate however, as stated above we have been unable to confirm previous studies suggesting a role in apoptosis resistance in AGS carcinoma cells *in vitro* [29]. It is likely that the gene targets of the BART miRNAs are linked to specific *in vivo* growth functions of both normal and malignant cells. One possible candidate is the difference in geometry between *in vivo* (3-dimensional) and *in vitro* (2-dimensional) growth. For example, it is possible that the BART miRNAs regulate signaling pathways that control tumor hypoxia *in vivo*, which could result in activation of a broad array of mitogenic, pro-invasive and pro-angiogenic genes [45–49]. It is also possible that the growth advantage provided by the miRNAs is a function of the interaction of the tumor cells with the surrounding environment *in vivo*. For example, the miRNAs may induce the tumor cells to express factors or cell surface changes that elicit growth promoting signals from the surrounding murine milieu in the form of soluble factors such as cytokines and/or recruited cells that potentiate tumor growth.

We have also seen no evidence that the BART miRNAs contribute to invasion or metastasis. Indeed we have observed a trend whereby the miRNAs may actually impede these processes since the rate of metastasis was lower for the BART+ tumors. This result could have arisen because the BART+ tumors grow more rapidly and may kill the mice before the tumors have time to metastasize. However, we also observed that the BART miRNAs have a negative impact on invasion, an important corollary of metastasis. It may simply be that the increased proliferation rate driven by the BART miRNAs marginally diverts the cellular metabolism away from the processes required for invasion and metastasis.

One possible concern with our studies is the high levels of expression we see for the BART miRNAs in tumors. We have previously estimated the BART miRNA copy numbers in biopsy material for the three tumor types studied here [50]. Even taking into account the presence of non-tumor cells in the biopsies and imprecision caused by technical difficulties in recovering the miRNAs from small biopsy samples it is clear that the mouse tumors are expressing the miRNAs at levels at least 10 fold higher than in the biopsies. However, the process driving this up regulation in the mice is physiologic, not for example an artifact of ectopic expression. This would suggest that there may be constraints on the miRNA copy level in the human host that are not imposed in the highly immune incompetent NSG mouse. One question that arises is with respect to the *in vivo* specificity of the up regulation of the BART miRNAs. Is it conceivable that if we could artificially drive the copy numbers high enough *in vitro*, we would see an effect on growth? However, in this scenario it is difficult to explain why the copy numbers are reduced again upon culturing the tumors *in vitro* since any growth advantage due to higher BART copy number should ensure their maintenance. This strongly argues that the selective growth advantage provided by the elevation of the BART miRNAs *in vivo* is not sustained *in vitro*.

In summary, therefore, we conclude that the BART miRNAs provide a significant growth advantage to infected tumor cells growing *in vivo*. This effect may be a combination of growth promoting and survival functions provided by the miRNAs.

## Methods

### Cell culture

The cell lines used in this study were: NPC line C666–1; BL cell line BL36 (gift of Dr. Jeff Sample); GaCa cell lines AGS and AGS-BX1 (gift of Dr. Lindsey Hutt-Fletcher); AGS cells transfected with oriP//EBNA1 vectors; and mouse tumor-derived explant lines. The GaCa cell lines and GaCa tumor-derived explant lines were cultured in Ham’s F-12 medium containing 10% fetal bovine serum (FBS), 2 mM sodium pyruvate, 2 mM glutamine, and 100 IU of penicillin-streptomycin. All other cells were maintained in RPMI 1640 medium with the same supplements. All adherent lines were passaged after trypsinization.

### Lentiviral infection and cell transfection

BL36, C666–1, AGS and AGS-BX1 cells were infected with the pGreenFire1-CMV, TR011VA-1 lentivirus (System Biosciences, Mountain View, CA), which expresses green fluorescent protein (GFP). GFP positive cells were sorted and collected by Fluorescence Activated Cell Sorting (FACS), and were subsequently cultured with appropriate medium. 2×10^6^ luciferase expressing AGS cells were transfected with 5 ug of plasmid DNA from either the oriP/EBNA1-EMPTY (p220) or oriP/EBNA1-BART vector (p3829) (gift of Dr. Bill Sugden) using an Amaxa nucleofector (Lonza), the V kit solution, and Program B-023. After 24 hr, transfected AGS (AGS-EBNA1-EMPTY and AGS-EBNA1-BART) were selected in F12 medium supplemented with 150ug/ml of hygromycin.

### Animal and histology studies

Female NOD.Cg-Prkdc^scid^ Il2rg^tm1Wjl^/SzJ (NSG) mice (the Jackson Lab, ME, USA) ages 6–8 weeks were housed and maintained under sterile conditions with free access to food and water. For carcinoma models, 2.5–7.5×10^5^ cells (C666–1, AGS, AGS-BX1, AGS-EBNA1-EMPTY and AGS-EBNA1-BART) resuspended in 50 ul of phosphate-buffered saline (PBS), were injected with a 25-gauge needle into the nasopharyngeal compartment of NSG mice under anesthesia [30]. For the lymphoma model, 2.5xl0^5^ BL36 cells in 100 ul PBS were injected i.v. via the tail vein into NSG mice. Disease progression was monitored based on overall health and bioluminescent imaging. Mice were intraperitoneally injected with luciferin followed by anesthesia with 3% isoflurane and subsequent measurement of bioluminescence using an IVIS 200 imaging system (Xenogen). Tumor burden (or volume) is presented as the radiance (photons per second per centimeter squared per steradian or p/s/cm2/sr) for each tumor by determining the photon emission/second of a given tumor within a radius encompassing 5% or greater of maximal signal intensity. The Kaplan-Meier survival curve analysis was conducted with the Prism program.

For histology analysis, tissue samples were fixed in 10% formalin buffer and stored in 75% ethanol prior to paraffin wax embedding, sectioning, hematoxylin and eosin (H&E) staining by the Animal Histology Core at Tufts Medical Center.

### *In vitro* tumor explants

A small proportion of tumor tissue was excised at surgical operation and rinsed with DPBS. The tissue was then transferred to a 100mm Petri dish with appropriate medium. For the carcinomas tissue was minced with sterile scalpels into smaller fragment (<2 mm) and pressed under a 3.0 µm PET membrane cell culture insert (BD Biosciences) followed by incubation at 37°C in a 5% CO_2_-humidified incubator. Carcinoma cells started to grow as a monolayer and attach to the dish in a few days. Occasionally, the dish also contained a few clumps of floating epithelial cells. For lymphoma explant cultures, the tissue was cut and minced into very small pieces in medium. The finely minced tissue in suspension was filtered with a 70 μm cell strainer to remove debris. The cells were pelleted by centrifugation at 1,500 rpm for 5 min and then resuspended in RPMI 1640 medium and grown as usual for suspension cell cultures.

### Total RNA and genomic DNA isolation

Tumor tissue was ground to a fine powder in a liquid nitrogen cooled mortar and pestle. For RNA, tissue powder was then extracted using Trizol (Invitrogen) according to the manufacturer’s instructions. For genomic DNA (gDNA), tissue powder was extracted with DNAzol (Invitrogen). Briefly, 1ml of DNAzol was added to the powder and the cell lysates were gently passed through pipettes several times for complete extraction. Insoluble material was removed by centrifugation at 10 000 *g* for 10 min at 25°C. The viscous supernatant was transferred into another tube and DNA was precipitated using 0.5 ml of 100% ethanol followed by inversion of the tubes several times. The tube was left at room temperature for 20 min and the DNA was pelleted by centrifugation at 14,000 g for 15 min at 4°C. The DNA was washed twice with 1ml 85% ethanol and then air dried. The genomic DNA was finally dissolved in 200 ul 8 mM NaOH and the pH adjusted to 7 by adding 20 uL 1 M HEPES buffer. RNA and DNA were directly purified from cell lines without using a mortar and pestle.

### miRNA expression profiling

EBV miRNA expression was measured using real-time multiplex reverse transcript (RT)-PCR [24] and is expressed relative to the ubiquitous small cellular RNA U1. Briefly, a panel of stem-loop RT primers specific for the 3′ end of each mature miRNA was pooled and used for RT of purified RNA with the TaqMan MicroRNA RT kit (Applied Biosystems), followed by TaqMan PCR using specific primers and probes to each miRNA. Calibration curves for estimating the EBV miRNA copy number were generated using serial dilutions of synthetic oligonucleotides for each miRNA. This assay has been used previously for profiling BART miRNA expression in infected human tissue, tumor biopsies and cell lines [22, 24]. Details of the assay including the lower limit of detection (≤10 copies of each miRNA) and linearity of the miRNA qPCR assay (up to ≥10^8^ copies) is described in [24]. For the human miRNAs mir-9, 26a and 34a, the same RT kit and human specific TaqMan MicroRNA assays were used (Life Technologies). The expression of all miRNAs in all experiments was normalized to the ubiquitous, small cell RNA U1.

### Quantitative gene and gDNA real-time PCR

mRNAs were measured by real time RT-PCR. A list of the primers used is provided in the supplemental material. RNA was reverse transcribed with an iScript cDNA synthesis kit (Biorad), followed by real-time PCR using IQ SYBR Green supermix (BioRad) and specific primers. EBER1 expression was assessed by TaqMan PCR as previously described. [51] The housekeeping genes GAPDH, actin and tubulin were used as internal control for normalization. Values relative to GAPDH are shown. The relative expression is expressed as 2^ΔCt^, where ΔCt = mean value Ct (gene of interest) − mean value Ct (GAPDH). For gDNA real time PCR, DNA was diluted 100-fold prior to PCR amplification with the IQ SYBR Green supermix and specific primers. All SYBR Green real time PCRs were performed on a Bio-Rad iCycler. The protocol was as follows: step 1, one cycle of 5 min at 95°C; step 2, 40 cycles of 15 s at 95°C and 1 min at 60°C; step 3, one cycle of 1 min at 95°C. Fluorescence was monitored at the end of each extension phase. After amplification, melting curves were generated to verify the specificity of amplification. Primers for amplification are listed in S3 Table.

### Calculation of the oriP/EBNA1 plasmid copy number per cell

To measure the oriP/EBNA1 plasmid copy number per cell in each AGS-EBNA1-EMPTY and AGS-EBNA1-BART tumor sample we divided the plasmid copy number by the number of cells in each sample. To determine how many cells there were, real time qRT-PCR was performed on each sample for the GAPDH mRNA. The Ct values were then converted to cell number from calibration curves (cell number versus RT-PCR signal (Ct)) of serial dilutions of the corresponding cell lines (AGS-EBNA1-EMPTY and AGS-EBNA1-BART).

The determine the total plasmid copy number in the tumor samples we performed gDNA real time PCR for the EBNA1 gene and the Ct values were read from standard curves (Ct versus copy number) generated by using serial 10-fold dilutions of a standard EBV (B95–8) containing suspension with 1.7 × 10^4^ DNA copies/μl (Advanced Biotechnologies).

### Growth curve and colony formation assay

For the growth curves, three separate cultures of 3×10^4^ cells were seeded in100 mm Petri dishes. The cell number was then counted in duplicate for each replicate using the Scepter 2.0 handheld automated cell counter (Millipore) at indicated time points. For the soft agar colony formation assays, 1×10^4^ cells were suspended in medium containing 0.4% agarose and overlaid onto a solidified layer of medium-containing 0.8% agarose in 6-well plates. Three separate cultures were set up for each cell line tested. After two-three weeks, colonies were counted and photographed in five fields for each replicate. For each assay 3 independently established BART positive cell lines and 2 independently established BART- lines were tested. The results were expressed as the means ± SEM of the combined data for measurements on all replicates of all the cell lines in a given group.

### Apoptosis analysis

For apoptosis sensitivity 4×10^5^ cells were plated onto 6 well plates. Three independent cultures were set up for each cell line. After overnight incubation, cells were treated with 80 uM of etoposide for 72 hr prior to harvest. Harvested cells were washed with Annexin V binding buffer and subsequently stained with Annexin V-APC (BD Bioscience) for 15 min, followed by fixation in 4% buffered paraformaldehyde at room temperature for 5 min. Apoptotic cells were analyzed in duplicate by flow cytometry and defined as positive for Annexin V-APC staining. The percentage of cells that had undergone apoptosis in response to etoposide was assessed by subtracting that of apoptotic cells in the untreated from the treated population (%Delta Annexin-V^+^).

### Matrigel invasion assays

*In vitro* invasion assays were carried out as described previously [52] using complete Matrigel (BD Biosciences). A total of 18 μg Matrigel at a concentration of 0.3 μg/μl was coated onto a FluoroBlok insert (BD Biosciences) with an 8 μm pore size membrane. The transwell insert was allowed to dry overnight at room temperature and rehydrated with 60 ul of serum-free Ham’s F-12 medium for 2 hr. Cells were seeded in triplicate at 5×10^4^ per transwell insert. 500 μl of media containing 5% FBS were added to the lower well of the assay chamber to act as a chemoattractant. After 24 hr, the transwell inserts were placed onto wells containing 4 μg/ml calcein AM in Hanks’ balanced salt solution and incubated for 30 min at 37°C in 5% CO_2_. Cells that had passed through the pores and reached the trans-side of the membrane were counted with the imaging software MetaXpress. The number of invading cells was averaged over triplicate wells and presented as the means ± SD.

### Ethics statement

All animal experiments described in this study were performed according to the Guidelines of the Tufts University Division of Laboratory Animal Medicine and were in accordance with the National Institutes of Health guide for the Care and Use of Laboratory Animals. Animal protocols were approved by the Tufts University Institutional Animal Care and Use Committee (protocol B2011–108).

## Supporting Information

S1 Fig Effect of ***in vivo*** tumor growth on colony forming potential and sensitivity to apoptosis. A. and B. A soft agar colony forming assay was performed on the parental (A) and explanted tumor (B) cells as described in Methods. C. and D. Parental (C) and explanted tumor (D) cells were treated with etoposide and the levels of apoptotic cells measured by Annexin V staining and FACS analysis. The % Delta Annexin V^+^ was assessed by subtracting the percent of apoptotic cells in the untreated from the treated population. All experiments were performed in triplicate and the value was average ± standard deviation. n.s. represents a not statistically significant difference (p > 0.05). * represents a significant statistical difference (p < 0.05).
(TIF)

S1 Table Cells used in EBV miRNA profiling. (DOCX)

S2 Table Xenograft models of EBV-positive carcinomas and lymphomas used in this study. (DOCX)

S3 Table Primers used in this study. (DOCX)
