# Supplementary figures and images for: The Epstein-Barr Virus Encoded BART miRNAs Potentiate Tumor Growth In Vivo
Source: PLoS Pathog. 2015 Jan 15;11(1):e1004561. doi: 10.1371/journal.ppat.1004561 (PMC4295875; doi:10.1371/journal.ppat.1004561)

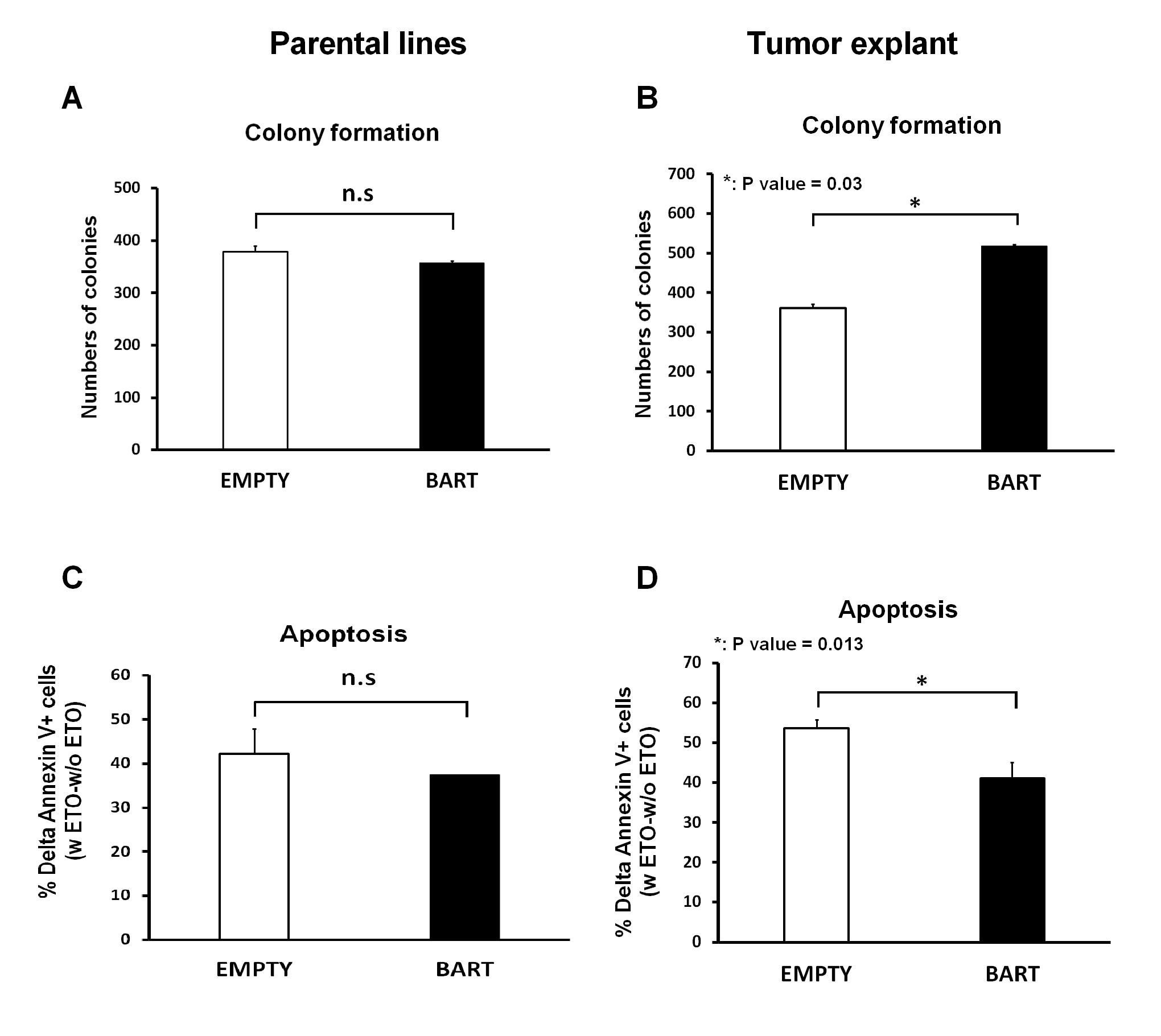

Supplement: S1 Fig — A. and B. A soft agar colony forming assay was performed on the parental (A) and explanted tumor (B) cells as described in Methods. C. and D. Parental (C) and explanted tumor (D) cells were treated with etoposide and the levels of apoptotic cells measured by Annexin V staining and FACS analysis. The % Delta Annexin V+ was assessed by subtracting the percent of apoptotic cells in the untreated from the treated population. All experiments were performed in triplicate and the value was average ± standard deviation. n.s. represents a not statistically significant difference (p > 0.05). * represents a significant statistical difference (p < 0.05). (TIF) [file ppat.1004561.s001.tif]
