# Supplementary material for: The Epstein-Barr Virus Encoded BART miRNAs Potentiate Tumor Growth In Vivo
Source: PLoS Pathog. 2015 Jan 15;11(1):e1004561. doi: 10.1371/journal.ppat.1004561 (PMC4295875; doi:10.1371/journal.ppat.1004561)
Supplement: S1 Table — (DOCX) [file ppat.1004561.s002.docx]

Supplemental Table S1. Cells used in EBV miRNA profiling.

| Parental lines | *In vivo* tumors (#) | *In vitro* explant lines (#) |
| --- | --- | --- |
| C666-1 | Primary(4), metastasis(4), flank(1) | Primary(1), metastasis ( 4) |
| AGS-BX1 | Primary(2), metastasis (5), flank(1) | Primary(2), metastasis (4) |
| BL36 | Lymph node(3), spleen(1), ovary(2), lung(1) | lymph node(1), spleen(1),ovary(1), lung(1) |
| AGS-EBNA1-BART | Primary (3), metastasis (2) | Primary (3) |
