# Supplementary material for: The Epstein-Barr Virus Encoded BART miRNAs Potentiate Tumor Growth In Vivo
Source: PLoS Pathog. 2015 Jan 15;11(1):e1004561. doi: 10.1371/journal.ppat.1004561 (PMC4295875; doi:10.1371/journal.ppat.1004561)
Supplement: S2 Table — (DOCX) [file ppat.1004561.s003.docx]

Supplemental Table S2. Xenograft models of EBV-positive carcinomas and lymphomas used in this study.

| Cell lines^1^ | Origin | Injection site | Mice with tumors/  total injected | Take rate % | Days to tumor (median) | Survival days (median) | No. with metastases | Organ involved (# of metastases) |
| --- | --- | --- | --- | --- | --- | --- | --- | --- |
| C666-1 | NPC | I.N. | 23/24 | 96% | 21 | 40 | 10 | .Ovary (1), spleen (1), liver (2) |
| AGS-BX1^2^ | GaCa | I.N. | 4/10 | 40% | 62 | 155 | 3(4) | Lung(2), Lymph node(1), Bone(1), uterus(1) |
| BL36 | BL | I.V. | *10/10 | 100% | 21 | 32 | N/D | Lymph node(4),Lung(2), spleen(1), ovary(2), bone marrow(1) |

1. Similar tumorigenic phenotypes were also observed for the breast adenocarcinoma cell lines MCF-7 and MDA-MB-231 (not shown). These results indicate that the murine nasopharyngeal epithelium is an excellent site for studying the growth, invasion and metastasis of human carcinomas.
2. Successful engraftment of immunosuppressed mice with AGS-BX1 has not been reported previously. Consistent with this mice injected subcutaneously with 10^7^ AGS-BX1 cells failed to develop tumors. In our hands 4 out of 10 mice injected nasopharyngeally (I.N.) with AGS-BX1 grew tumors, with median time to tumor appearance of 62 days and median lifespan of 155 days. Metastatic dissemination was observed in 3 of the mice. Metastasized AGS-BX1 cells accumulated in the lung capillary and proceeded to either invade the alveolar wall or localize at the air spaces. The invasion of tumor cells in organs e.g. kidney is often accompanied with massive infiltration of histiocytes reflecting an inflammatory responses and that this model is suitable to study GaCa *in vivo*.
3. BL36 cells gave rise to progressively growing tumors in all 10 animals. The disease onset became evident at approximately 21 Days followed by rapid deterioration of the animals, which often showed signs of lymphoma attack such as tumor cachexia and hind limb paralysis. Tumors were found in many organs but were most common in lymph nodes, spleen, lung and bone. Pathologically, lymphoma was composed of monomorphic BL cells predominantly of small to medium size with round or irregular nuclei. The neoplastic cells often markedly packed the sinuses of lymphoid organs i.e. spleen, interspersed with nonfunctional lymphocytes and histiocytes.
