# Supplementary material for: The Epstein-Barr Virus Encoded BART miRNAs Potentiate Tumor Growth In Vivo
Source: PLoS Pathog. 2015 Jan 15;11(1):e1004561. doi: 10.1371/journal.ppat.1004561 (PMC4295875; doi:10.1371/journal.ppat.1004561)
Supplement: S3 Table — (DOCX) [file ppat.1004561.s004.docx]

Table S3. Primers used in this study.

| Human GAPDH (PrimerBank ID 7669492a3): | F:CATGAGAAGTATGACAACAGCCT  R: AGTCCTTCCACGATACCAAAGT |
| --- | --- |
| Human e-cadherin (CDH1) (PrimerBank ID 4757960a3): | F:CCCACCACGTACAAGGGTC  R:ATGCCATCGTTGTTCACTGGA |
| Human snai1(PrimerBank ID 301336132b1): | F:TCGGAAGCCTAACTACAGCGA  R:AGATGAGCATTGGCAGCGAG |
| LMP1: | F:AGCCCTCCTTGTCCTCTATTCCTT  R:ACCAAGTCGCCAGAGAATCTCCAA |
| EBNA1: | F: GGTCGTGGACGTGGAGAAAA  R: GGTGGAGACCCGGATGATG |
| EBER1: | F: ACCGAAGACGGCAGAAAGC  R: CCTACGCTGCCCTAGAGGTTT  Probe: 6-carboxy-fluorescein-ACAGACACCGTCCTCACCACCCG-6-carboxymethylrhodamine. |
